# Endothelial Cell Morphology Regulates Inflammatory Cells Through MicroRNA Transferred by Extracellular Vesicles

**DOI:** 10.3389/fbioe.2020.00369

**Published:** 2020-05-19

**Authors:** Jiaqi Liang, Shuangying Gu, Xiuli Mao, Yiling Tan, Huanli Wang, Song Li, Yue Zhou

**Affiliations:** ^1^Shanghai Jiao Tong University Affiliated Sixth People’s Hospital, School of Biomedical Engineering, Med-X Research Institution, Shanghai Jiao Tong University, Shanghai, China; ^2^Department of Bioengineering, Department of Medicine, University of California, Los Angeles, Los Angeles, CA, United States

**Keywords:** extracellular vesicles, microtopology, vascular inflammation, monocytes, miR-10a

## Abstract

Vascular inflammation plays an important role in the pathogenesis and the development of cardiovascular diseases such as arteriosclerosis and restenosis, and the dysfunction of endothelial cells (ECs) may result in the activation of monocytes and other inflammatory cells. ECs exhibit an elongated morphology in the straight part of arteries but a cobblestone shape near the pro-atherogenic region such as branch bifurcation. Although the effects of hemodynamic forces on ECs have been widely studied, it is not clear whether the EC morphology affects its own function and thus the inflammatory response of monocytes. Here we showed that elongated ECs cultured on poly-(dimethyl siloxane) membrane surface with microgrooves significantly suppressed the activation of the monocytes in co-culture, in comparison to ECs with a cobblestone shape. The transfer of EC-conditioned medium to monocytes had the same effect, suggesting that soluble factors were involved in EC–monocyte communication. Further investigation demonstrated that elongated ECs upregulated the expression of anti-inflammatory microRNAs, especially miR-10a. Moreover, miR-10a was found in the extracellular vesicles (EVs) released by ECs and transferred to monocytes, and the inhibition of EV secretion from ECs repressed the upregulation of miR-10a. Consistently, the inhibition of miR-10a expression in ECs reduced their anti-inflammatory effect on monocytes. These results reveal that the EC morphology can regulate inflammatory response through EVs, which provides a basis for the design and the optimization of biomaterials for vascular tissue engineering.

## Introduction

Atherosclerosis is the major cause of cardiovascular diseases (Momiyama et al., 2005). Although atherosclerosis develops through multiple discrete stages (Puri et al., 2011), vascular inflammation is involved in the whole process of disease progression (Ross, 1999; Davignon and Ganz, 2004). Healthy endothelial cells (ECs) control vascular tone, limit vascular smooth muscle cell (VSMC) proliferation, inhibit leukocyte adherence, and block thrombosis (Landmesser et al., 2004). However, a variety of vascular injuries destroy the functions of the endothelium from protecting the vessel wall (Vanhoutte et al., 2009). Endothelial injury represents a major initiating step in the pathogenesis of vascular disease and atherosclerosis (Boos et al., 2007), followed by the activation and the recruitment of circulating monocytes to the region of vascular injury or infection (Ley et al., 2007). After their entry into the vessel wall, the monocytes differentiate into macrophages, which contributes to inflammatory response to neutralize invading pathogens, repair tissue damage, or activate other immune cells (Shi and Pamer, 2011). In addition to EC injury, there is evidence that the flow pattern also regulates EC morphology and activation. ECs in the straight part of an artery have an elongated morphology and align in the direction of laminar flow. In contrast, in the branched area and the curved part of artery, the formation of vortex and disturbed flow activated ECs and promoted inflammatory signaling (Li et al., 2005), and the ECs in these areas exhibit a cobblestone shape. However, whether the EC morphology *per se* has direct effects on inflammatory cells is not well-understood.

Monocyte activation contributes to the pathogenesis of various inflammatory conditions and atherosclerosis (Woollard and Geissmann, 2010). Such inflammatory response is regulated by various signals in the microenvironment, such as microbial products, cytokines, and microRNAs. It has been reported that circulating microRNAs exert great influence in modulating monocyte/macrophage phenotype and function in the process of vascular inflammation (O’Connell et al., 2007; Tili et al., 2007; Ono et al., 2011). Vascular inflammation is an important early event in atherogenesis, where many microRNAs are involved, including miR-10 (Fang et al., 2010), miR-17, miR-31 (Suarez et al., 2010), miR-92 (Wu et al., 2011), miR-155 (Zhu et al., 2011), miR-221, and miR-222 (Liu et al., 2012; Chen et al., 2019). Circulating miRNAs are not only biomarkers for disease but also serve as cell-to-cell messengers (Hergenreider et al., 2012; Yamakuchi, 2012).

Since naked RNAs are easy to be degraded by ribonuclease, microRNAs in circulation can exist either in protein binding form or enclosed in extracellular vesicles (EVs). The transfer of microRNAs in EVs mediating through interactions between the wide varieties of cell types in the cardiovascular system (Das and Halushka, 2015) has now been reported in cardiovascular systems and disease. ECs can modulate myeloid inflammatory responses through the secretion of EVs containing anti-inflammatory miRNAs (Njock et al., 2015).

In addition to biochemical signals, biophysical factors in the microenvironment may cause significant changes in the gene expression and the cellular behavior to execute regulatory function in diverse vascular events. For example, nano/micro-topographic cues can significantly affect the gene expression of human vascular ECs, which can also affect the progress of cardiovascular diseases (Biela et al., 2009; Gasiorowski et al., 2010). In addition, biophysical cues, in the form of parallel microgroove on the surface of cell-adhesive poly-(dimethyl siloxane) (PDMS) substrates, can replace the effects of small-molecule epigenetic modifiers and significantly improve cell reprogramming efficiency (Downing et al., 2013). However, it is not clear whether micro/nano-topography regulates cell–cell communications through EVs.

Here we investigated whether and how a specific pattern on culture substrates could induce pronounced changes in EC morphology and functions through microRNA-enclosing EVs to modulate inflammatory cells.

## Materials and Methods

### Fabrication and Characterization of the Culture Substrate

Microfabrication technique was used to fabricate PDMS membranes with desired surface topography (10 μm in width, 3 μm in depth, and 10 μm spacing between each microgroove). PDMS was prepared according to the manufacturer’s instruction (Dow Corning, United States), spin-coated onto the patterned silicon wafers to achieve the desired thickness, degassed under vacuum, and cured at 75°C for 1.5 h. The micropatterned membranes were removed from the template, cut to appropriate dimensions and thoroughly cleaned by sonication, treated with Plasma Prep III (11050Q-AX) to enhance the surface hydrophilicity, and coated with 2% gelatin for 1.5 h to promote cell attachment. The images of surface topography of the micropatterned PDMS membranes were collected by using scanning electron microscopy (SEM; JEOL JSM-5600, Japan).

### Cell Culture

Human umbilical vein endothelial cells (Sciencell, United States) were cultured in ECM medium (Sciencell, United States) with 5% fetal bovine serum (FBS), 1% penicillin/streptomycin, and 1% endothelial cell growth supplement at 37°C in a humidified 5% CO_2_ incubator. The cells were seeded on the PDMS membrane and cultured to reach confluence before proceeding to the experiments. Monocyte cell line THP-1 (human acute monocytic leukemia cell line) was obtained from the American Type Culture Collection and cultured in RPMI 1640 medium supplemented with 1% penicillin/streptomycin, 10% fetal bovine serum, L-glutamine, and 0.05 mM β-mercaptoethanol. The cells were grown to a density of 1 × 10^6^cells/ml and treated with 50 ng/ml lipopolysaccharide (LPS) for 2 h for activation.

### Co-culture

Monocytes and ECs co-culture was carried out in a Transwell^TM^ system purchased from Thermo (Cat# Nunc, 140660). The PDMS membrane was attached to the outer surface at the bottom of the Transwell insert, with the ECs facing down to the monocyte culture in the lower well, leaving an ^˜^1-mm space in between. Before co-culturing with ECs, the monocytes were stimulated with LPS at a concentration of 50 ng/ml for 2 h and washed thoroughly to remove the additional LPS. The total volume of the medium in the co-culture system was 2 ml, comprising of 1 ml of EC medium and 1 ml of RPMI medium (for monocyte). To make sure that the EVs that we extracted were from ECs and not from the FBS, exosome-depleted FBS (System Biosciences, LLC, United States) was used in the co-culture and trans-medium system.

### Immunofluorescence Staining

The cells were fixed with 4% paraformaldehyde (Electron Microscopy Sciences, United States) and blocked with 10% BSA-PBST (Merck, United States). For actin–cytoskeleton staining, the samples were incubated with fluorescein-isothiocyanate-conjugated phalloidin (FITC; AAT Bioquest, United States) for 1 h. For immunostaining, primary antibodies were incubated overnight at 4°C, followed by 1 h of incubation with proper secondary antibodies in the dark. The nuclei were stained with 4’,6-diamidino-2-phenylindole (DAPI; Beyotime Biotechnology, China). The microscopic images were acquired with confocal laser scanning microscope TCS SP5II (Leica, Germany).

### Nanoparticle Tracking Analysis

After culturing for 24 h, the EVs were isolated from the EC culture medium by ultracentrifugation. Briefly, cell culture medium was collected and centrifuged at 300 *g* for 10 min and at 2,000 *g* for 10 min to remove cell debris. Then, the supernatant was centrifuged at 100,000 *g* for 70 min (Beckman XE-90, United States) to separate the EVs and the EV-depleted medium. Finally, the EVs were resuspended in 1 ml Dulbecco’s phosphate-buffered saline. The size of the EVs was analyzed by the manufacturer’s default software of Zeta View 8.04.02 SP2 (Bachurski et al., 2019).

### EV Uptaking

The EVs extracted from the conditioned medium of ECs by ultracentrifugation were labeled with I-135 according to a previously published method (Zhang et al., 2016) and added into the monocyte medium. After 2 h, the intensity of the radiation from the monocytes was measured with a scintillation counter and expressed as counts. Normal culture medium was used as blank for background reading.

### RNA Isolation and qRT-PCR Analysis

The total RNA and microRNAs in the cells were isolated using TRIzol^®^ plus RNA Purification (Invitrogen, United States) and miRcute miRNA isolation kit (Tiangen, China), respectively, following the manufacturer’s instruction. For qRT-PCR, cDNA of mRNA synthesis was performed using the FastQuant RT kit (with gDNase) (Tiangen, China), and the mRNA level was measured using SuperReal PreMix Plus (SYBR Green) (Tiangen, China). Here the GAPDH was used as a normalization control. The sequences of the primers used for real-time PCR are listed in Table 1.

**TABLE 1:** Primer sequences for mRNA quantitation in qRT-PCR.

Primers	Forward primer (5’–3’)	Reverse primer (5’–3’)
GAPDH	5’ GGG AAG GTG AAG GTC GGA GT	5’ GGG GTC ATT GAT GGC AAC A
MAP3K7	5’ TGA CTC CTC CAT AGC ATT GT	5’ CAT CAA GCC TTA GCA TTC AC
IL-6	5’ GCA CCT CAG ATT GTT GTT G	5’ AAA TAG TGT CCT AAC GCT CA
TNF-α	5’ AGT CG GGC AGG TCT ACT TT	5’ CGT TTG GGA AGG TTG GAT GT
IL-1β	5’ GCT GGC AGA AAG GGA ACA GA	5’ GCA GTT GGG CAT TGG TGT AG
IL-10	5’ CCA AGA GAA AGG CAT CTA CA	5’ GGG GGT TGA GGT ATC AGA G

Total Exosome RNA and Protein Isolation Kit (Invitrogen, United States) was used for microRNA isolation from EVs. The EVs were dissolved in a 37°C pre-warmed denaturing solution. The microRNAs were extracted by a mixture of phenol/chloroform solution and precipitated from the aqueous phase by ethanol, followed by a series of wash and elution steps. The purified microRNAs were finally dissolved in the elution solution provided in the kit before the subsequent cDNA first-strand synthesis experiments. The cDNAs of the microRNAs were synthesized with miRcute miRNA First-Strand cDNA Synthesis Kit, and the quantitative real-time PCR was performed with miRcute miRNA qPCR Detection Kit (SYBR Green) (Tiangen, China) according to the manufacturer’s instruction. RNU6-2 was used as a normalization control in all microRNA measurements. The relevant primers are listed in Table 2. The qRT-PCR was run on Applied Biosystems 7900HT Fast Real-Time PCR System (ABI, United States). The qRT-PCR data were analyzed using the comparative 2^–ΔΔCT^ method.

**TABLE 2:** Primer sequences for microRNA quantitation in qRT-PCR.

Primers	Forward primer
hsa-miR-10a-5p	5’ CAC AAA UUC GGA UCU ACA GGG UA
hsa-miR-126-3p	5’ CGC AUU AUU ACU CAC GGU ACG A

### Enzyme-Linked Immunosorbent Assay

The total of IL-6 protein in monocyte culture media was quantified by enzyme-linked immunosorbent assay (ELISA) (R&D, United States). The operating procedure follows the manufacturer’s recommendations.

### EV Secretion Inhibition

N-SMase Spiroepoxide inhibitor is a potent and selective irreversible inhibitor of neutral sphingomyelinase (N-SMase), and it has been used to inhibit exosome release (Devhare et al., 2017). To inhibit EV production, N-SMase Spiroepoxide inhibitor (C_20_H_30_N_2_O_5_, Santa Cruz, United States) was used at a concentration of 2.5 μm to treat the ECs for 24 h. Dimethyl sulfoxide (Invitrogen, United States) was used as the solvent control.

### MicroRNA Inhibition

ECs were seeded on the flat or microgrooved PDMS membranes for 24 h, and then miR-10a inhibitor or negative control was added into the medium at a concentration of 100 nM, following the manufacturer’s protocol. The trans-medium system was applied to culture monocytes for 24 h. The micrOFFTM hsa-miR-10a-5p inhibitor, the negative control of micrOFFTM inhibitor, and the FECT^TM^ CP Transfection Kit (Ribobio, China) were used in this experiment.

### Western Blotting

For Western blotting analysis of the exosome marker, the exosomes were isolated using Total Exosome Isolation Kit (Invitrogen, United States), according to the manufacturer’s instruction. Exosome samples were directly denatured with the loading buffer, separated by sodium dodecyl sulfate-polyacrylamide gel electrophoresis (SDS-PAGE), and transferred to polyvinylidene fluoride membranes. The membrane was blocked in 5% non-fat milk and incubated with primary antibody against CD63 (Thermo Fisher Scientific, United States) at a concentration of 2 μg/ml and HRP conjugated goat anti-mouse IgG (Jackson immunoResearch Laboratories, Inc., United States) as secondary antibody. Then, blots were incubated using Immobilon Western Chemiluminescent HRP substrate (Merck Millipore, United States) and visualized and recorded on Tanon 5200 chemiluminescence imaging analysis system (Tanon Science & Technology Co Ltd., China).

### Statistical Analysis

Unless otherwise indicated, data represent the mean of at least three independent experiments and error bars represent the standard error of the mean. Pairwise comparisons were made using Student’s *t*-test. A comparison of three or more groups was performed using ANOVA. Differences between groups were then determined using Tukey’s *post-hoc* test. For all cases, *p* < 0.05 were considered as statistically significant. In all figures, the asterisks represent *P <* 0.05. GraphPad Prism 6.0 software was used for all the statistical evaluations.

## Results

### Elongated ECs Suppressed Inflammatory Response in Co-cultured Monocytes

**FIGURE 1:**
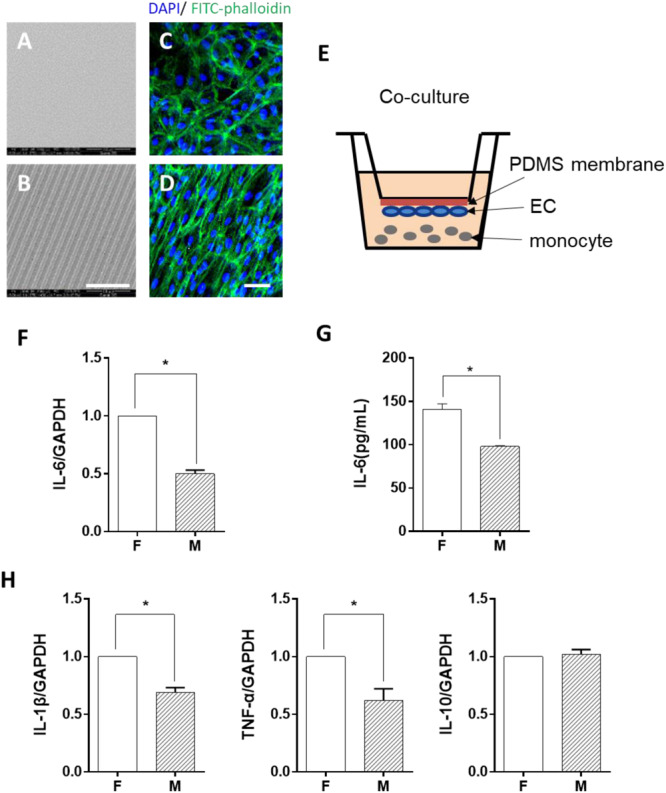
Morphology-modified endothelial cells (ECs) inhibited the inflammatory cytokine expression in monocytes in the co-culture system. **(A,B)** Scanning electron micrograph image of the flat (F) and microgrooved (M) PDMS membranes, showing the parallel microgrooves with 10 μm in width and 3 μm in depth. Scale bar is 100 μm. ECs were seeded on flat **(C)** or microgrooved **(D)** poly-(dimethyl siloxane) (PDMS) membrane and cultured for 24 h. The F-actin was stained by FITC-phalloidin (green). The cell nucleus was stained by 4’,6-diamidino-2-phenylindole (blue). Scale bar is 75 μm. **(E)** Schematic of the EC–monocyte co-culture system using a transwell apparatus. **(F,H)** LPS-treated monocytes were co-cultured with ECs on either flat or microgrooved PDMS membrane for 24 h. The expression level of inflammatory cytokines (IL-6, IL-1β, TNF-α, and IL-10) in the monocytes was assessed by qRT-PCR. **(G)** The secreted protein level of IL-6 was determined by ELISA. Data were normalized to GAPDH expression. All the experiments were repeated for at least three times, *p < 0.05.

ECs were seeded on either flat PDMS membrane (Figure 1A) or micropatterned PDMS with 10-μm parallel microgrooves (Figure 1B), as shown in the SEM image. The morphology of the ECs was demonstrated by both FITC-phalloidin (green) and DAPI (blue) (Figures 1C,D) staining. Phalloidin staining of the stress fibers indicated that the microgrooved substrates had a pronounced effect on EC cytoskeleton and morphology. In general, cells grown on microgrooved PDMS membranes exhibited a more elongated morphology, following the guide of parallel microgrooves, compared to those cultured on flat PDMS membrane, and the stress fibers were also parallel to the axis of the microgrooves. LPS was used to treat the monocytes, which induced a robust expression of IL-6, IL-1β, and TNF-α. The EC co-culture (Supplementary Figure S1) significantly decreased the expression of these inflammatory genes. This is consistent with the precious findings that ECs suppress inflammatory responses. In addition, ECs on microgrooves showed more suppressive effects. Therefore, in the rest of the studies, we focused on the effects of EC morphology on monocytes following LPS stimulation.

To determine whether ECs cultured on microgrooved PDMS membranes could modulate the inflammatory cells, THP-1 cell line was used as a pro-inflammatory monocyte model in this study. The monocytes were treated with LPS for 2 h and were co-cultured with ECs in the system illustrated in Figure 1E. After 24 h, the expression level of the inflammatory cytokines IL-6, IL-1β, and TNF-α was assessed (Figures 1F,H). Compared with ECs on the flat PDMS membrane, the mRNA expression level of IL-6, IL-1β, and TNF-α was significantly decreased. In addition, the amount of the soluble IL-6 protein in the culture medium was further verified by ELISA (Figure 1G), showing the same trend as its mRNA expression level. The decrease in the inflammatory cytokine level suggested that the inflammatory response of the monocytes was significantly suppressed by ECs grown on the microgrooved PDMS membrane.

### EC Morphology Regulated Monocytes Through Soluble Factors

**FIGURE 2:**
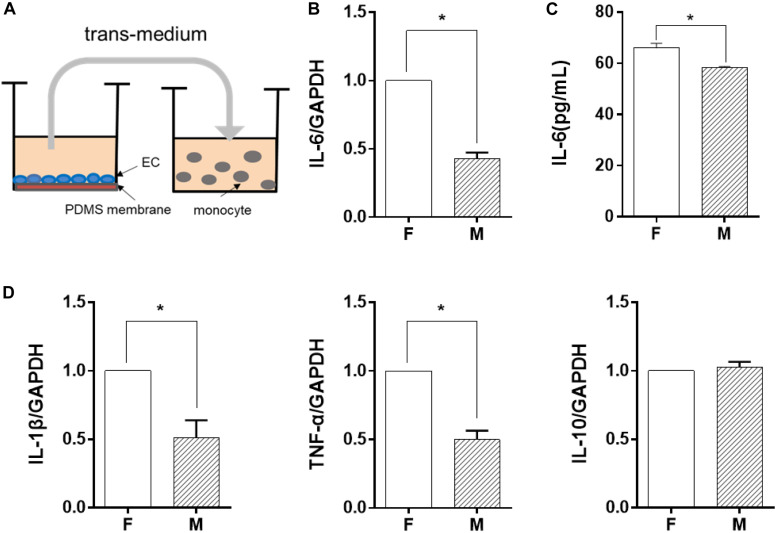
Endothelial cells (EC) morphology-regulated monocytes in a trans-medium system. ECs were cultured on flat (F) or microgrooved (M) membrane for 24 h, and the conditioned medium was collected and transferred to treat lipopolysaccharide-activated monocytes for 24 h. **(A)** Schematic of the trans-medium system. **(B,D)** The expression level of inflammatory cytokines (IL-6, IL-1β, TNF-α, and IL-10) was assessed by qRT-PCR. Data were normalized to GAPDH expression. **(C)** The protein level of IL-6 was determined by ELISA. All the experiments were repeated at least three times, *p < 0.05.

In order to identify the possible regulator in the EC-conditioned medium, which altered the monocyte inflammatory response, we used a trans-medium system (as shown in Figure 2A). The purpose was to verify whether ECs could exert a unidirectional regulatory effect on monocyte inflammation. The EC-conditioned medium was transferred to LPS-activated monocytes. The monocytes were treated for 24 h and the mRNA expression level of the inflammatory cytokines IL-6, IL-1β, TNF-α, and IL-10 was assessed (Figures 2B,D). Similar to the results demonstrated in Figure 1, compared with ECs on the flat PDMS membrane, the level of IL-6, IL-1β, and TNF-α was significantly decreased, except for IL-10. Therefore, we focused on IL-6 expression in the studies thereafter. The level of soluble IL-6 protein in the culture medium determined by ELISA (Figure 2C) also showed a significant decrease, which was consistent with its mRNA expression level. The decrease in the inflammatory cytokine level indicated that the inflammatory response of the monocytes was significantly suppressed by the factors secreted from the ECs grown on the microgrooved PDMS membrane.

### miR-10a Expression in Monocytes Was Affected by Morphology-Modified ECs

**FIGURE 3:**
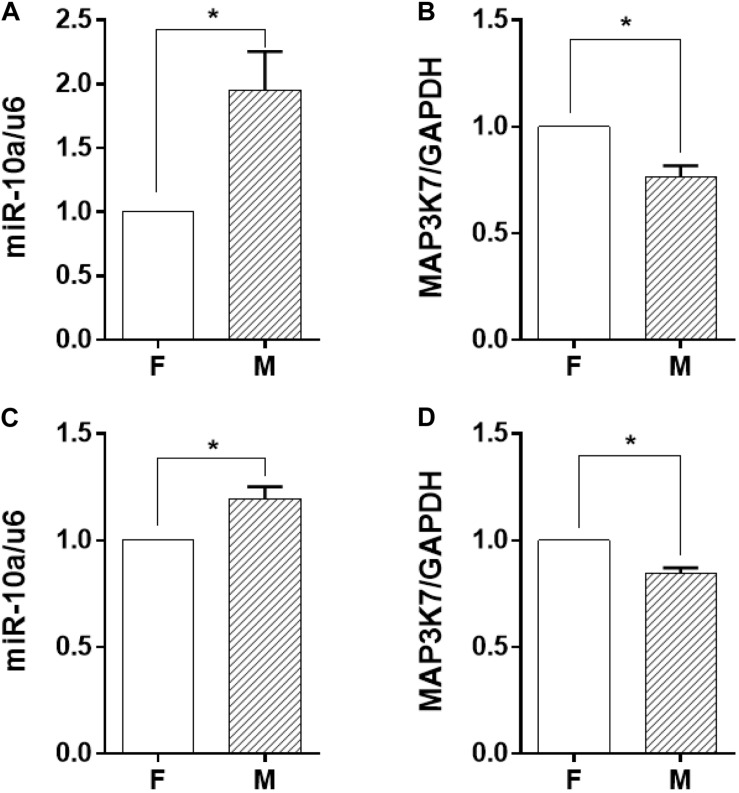
The miR-10a expression in monocytes was affected by morphology-modified endothelial cells. In both co-culture (panels in the upper row) and trans-medium (panels in the lower row) systems, the expression level of miR-10a in monocytes **(A,C)** was analyzed. Data were normalized to U6 expression. **(B,D)** The mRNA expression level of MAP3K7 in monocytes was analyzed by qRT-PCR. Data were normalized to GAPDH expression. All the experiments were repeated three times, *p < 0.05.

Among the soluble factors in the conditioned medium, microRNAs have been reported to have a great regulatory effect on cellular inflammatory response, and miR-10a has an inhibitory effect on IL-6 expression (Njock et al., 2015). Therefore, we explored further to determine whether miR-10a was the upstream regulator of the inflammation-suppressed status of the monocytes. The expression level of miR-10a in monocytes was analyzed in both co-culture and trans-medium systems. In the co-culture system, the miR-10a expression level in monocytes was elevated while they were co-cultured with ECs on the microgrooved PDMS membrane (Figure 3A). Consistently, the mRNA expression of MAP3K7, known as miR-10a direct target (Fang et al., 2010), was suppressed in monocytes (Figure 3B). Similarly, while only the EC-conditioned medium was used to treat the monocytes, the expression level of miR-10a in monocytes was elevated by the conditioned medium of ECs on the microgrooved PDMS membrane in comparison to that on the flat PDMS membrane (Figure 3C), and the mRNA expression of MAP3K7 in monocytes was suppressed accordingly (Figure 3D). Both the co-culture and the trans-medium experiments showed that miR-10a expression in monocytes was affected by morphology-modified ECs.

### miR-10a Expression in Monocytes Was Affected by EC-Secreted EVs

**FIGURE 4:**
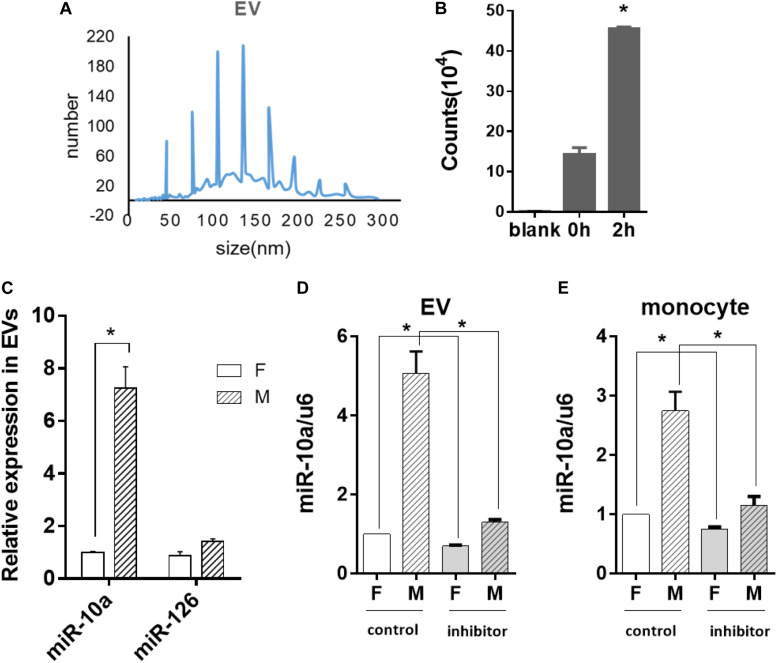
Morphology-modified endothelial cells (ECs) altered the miR-10a expression in monocytes through extracellular vesicles (EVs). ECs were cultured on flat or microgrooved poly-(dimethyl siloxane) membranes, and the conditioned medium of ECs was collected to extract EVs. **(A)** Nanoparticle tracking analysis was applied to analyze the size of EVs. **(B)** EVs extracted from the conditioned medium of ECs were uptaken by monocytes. **(C)** The expression level of miR-10a and miR-126 in EVs was determined by qRT-PCR. **(D)** N-SMase Spiroepoxide inhibitor was added to the cell medium to treat the ECs for 24 h. The expression level of miR-10a in EVs from the conditioned medium of ECs was determined. **(E)** The conditioned medium of ECs was transferred to culture monocytes for 24 h. The expression level of miR-10a in monocytes was determined by qRT-PCR. Data were normalized to U6 expression. Dimethyl sulfoxide was used as control. Inhibitor refers to N-SMase Spiroepoxide inhibitor. All the experiments were repeated three times, *p < 0.05.

Since miR-10a expression in the monocytes was affected by soluble factors in the conditioned medium, we further explored whether miRNA could be transferred by EVs. Thus, EVs from the culture medium of the ECs were isolated and analyzed. Nanoparticle tracking analysis demonstrated that the isolated EVs were 40–200 nm in diameter (Figure 4A), which is consistent with the size of small to medium EVs. Since EVs are heterogeneous and exosome is one of the major subpopulations in EVs, we performed exosome isolation and verified the presence of exosomes in isolated EVs by transmission electron microscopy and Western blotting analysis of CD63 expression (Supplementary Figure S2).

In order to confirm whether the EVs were uptaken by monocytes, the EVs extracted from the EC-conditioned medium were labeled with I-135 and added into the culture medium of the monocytes. Normal culture medium was used as blank control. The intensity of the radiation from the monocytes incubated with I-135-labeled EVs within 2 h was measured with a scintillation counter and expressed as counts. The result indicated that the EVs were uptaken by monocytes immediately and increased gradually (Figure 4B). Then, we analyzed the expression level of microRNAs in EVs. The miR-10a showed a robust increase in EVs (Figure 4C). In contrast, there was no significant change of another inflammatory-modulator miR-126.

If the anti-inflammatory effect of the morphology-modified ECs was indeed passed by EVs enclosing a specific microRNA, the inhibition of EV secretion from the ECs should block the change of miR-10a in monocytes. N-SMase Spiroepoxide inhibitor was used to block the formation and the release of the EVs from the ECs. To avoid the possible side effects of the inhibitor on the monocytes, a trans-medium system was used to culture the cells separately. The ECs were treated with the inhibitor for 24 h. Then, the conditioned medium was transferred to the monocytes for an additional 24 h. Upon the inhibition of EV secretion, the level of miR-10a was significantly decreased in EVs (Figure 4D) and monocytes (Figure 4E). In order to confirm that the monocyte response was affected by miR-10a in EVs, we used EVs only and EV-excluded medium (both from flat and microgrooved surfaces) to culture LPS-stimulated monocytes (Supplementary Figure S3). We found that EVs from ECs cultured on the microgrooved surface significantly inhibited the inflammatory response of the monocytes, while EV-excluded medium showed no significant change. These results confirmed that monocyte response was affected by miR-10a in EVs, not random miR-10a in trans-medium.

### The Inflammatory Response of the Monocytes Was Inhibited by miR-10a Suppression in ECs

**FIGURE 5:**
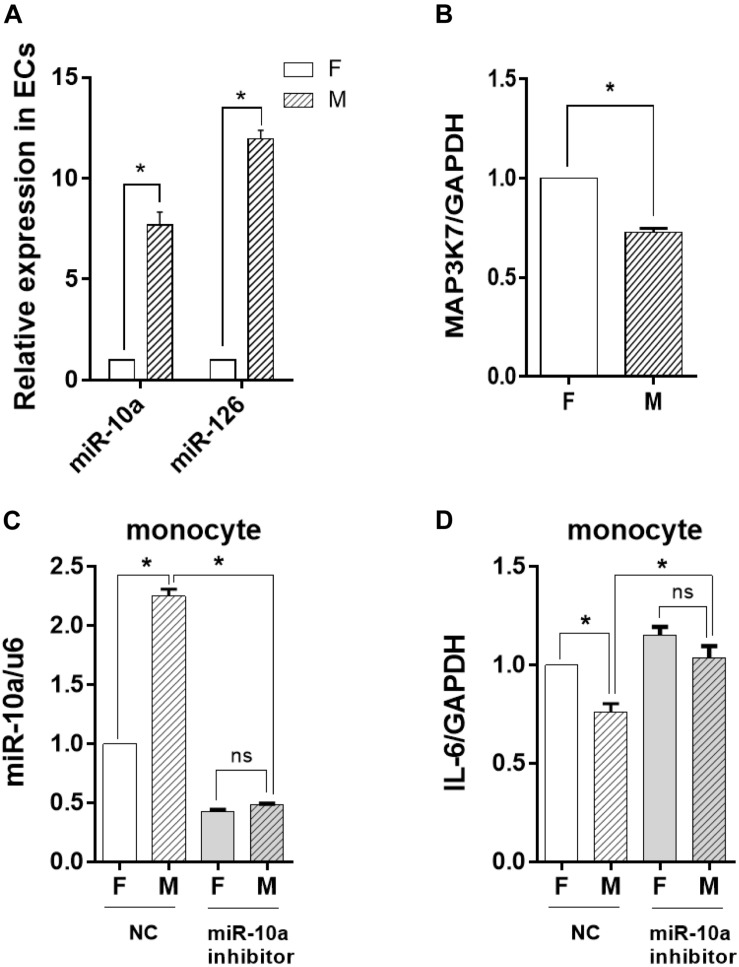
The inflammatory response of the monocytes was inhibited by miR-10a from morphology-modified endothelial cells (ECs). **(A)** The expression level of miR-10a and miR-126 in ECs cultured on flat (F) or microgrooved (M) poly-(dimethyl siloxane) membranes was determined by qRT-PCR. **(B)** The expression level of MAP3K7 in ECs was determined by qRT-PCR. ECs were treated with miR-10a inhibitor for 24 h. The conditioned medium of ECs was transferred to culture the LPS-treated monocytes for 24 h. **(C)** The expressions level of miR-10a in monocytes was determined. **(D)** The expression level of IL-6 was determined. NC refers to negative control. All the experiments were repeated three times, *p < 0.05 (ns, non-significant).

To determine whether the change of the EV-microRNA expression was sourced from ECs, we directly examined the level of miRNAs in ECs. The results in Figure 5A showed that the change of miRNA levels in ECs was consistent with those in EVs, i.e., miR-10a and miR-126 in ECs were also significantly induced. This result was confirmed by checking the mRNA level of MAP3K7, a miR-10a target gene, which had a significant decrease in ECs cultured on a microgrooved PDMS membrane (Figure 5B).

To investigate the effects of EC miRNA on miRNA and cytokine expression in monocytes, miR-10a inhibitor was applied to ECs cultured on a flat or a microgrooved surface. After 24 h, the EC-conditioned medium was transferred to culture the LPS-treated monocytes for additional 24 h. qRT-PCR analysis demonstrated that the expression level of miR-10a in monocytes decreased significantly and there was no more significant difference between ECs cultured on a flat surface and those cultured on a microgrooved membrane (Figure 5C). At the same time, the expression level of IL-6 in the monocytes was upregulated after inhibiting miR-10a, and also there was no more significant difference between ECs on a flat surface and those on a microgrooved surface (Figure 5D).

## Discussion and Conclusion

The pathogenesis and the progression of a lot of cardiovascular diseases such as atherosclerosis and thrombosis are accompanied by an inflammation response (Gistera and Hansson, 2017). In the blood vessel, the athero-prone regions are usually near the bifurcation of the arteries, where the hemodynamic forces are different from those in the straight part of the artery due to the blood flow (Wang et al., 2016). In this athero-prone lesion area, ECs show a cobblestone morphology in contrast to the elongated ECs in the straight part of the artery. Our study provides a direct evidence that this EC morphology change in the athero-prone area may upregulate microRNAs that can be transferred to monocytes *via* EVs and thus induce inflammatory signals.

MicroRNAs have long been implicated as key regulators of inflammatory responses in monocytes or macrophages (Fish and Cybulsky, 2012; Sun et al., 2013). In addition, the fluctuations in shear stress contribute to microRNA-mediated epigenetic regulation on the function of ECs, which are essential for the maintenance of vascular homeostasis (Ando and Yamamoto, 2011). A recent study identified miR-10a as a flow-responsive microRNA in ECs *in vitro* (Davignon and Ganz, 2004), and miR-10a could be induced by high shear stress as an athero-protective mechanism (Neth et al., 2013). Another study revealed that the expression of endothelial miR-10a was lower in the athero-susceptible regions of the inner aortic arch and the aorta–renal branches than that in other regions (Fang et al., 2010). Interestingly, the physical effect of the microgroove surface on EC morphology is similar to laminar shear stress, and consistently, elongated ECs have a higher level of miR-10a, suggesting that EC morphology change is sufficient to induce miR-10a expression.

Previous studies have demonstrated that miR-10a acts as a post-transcriptional modulator of the IκB/NF-κB signaling pathway by inhibiting MAP3K7 and βTRC (Fang et al., 2010; Njock et al., 2015). The increased proteolysis of IκBα and nuclear p65 in atherosclerotic susceptible areas resulted in a significantly up-regulated expression of the inflammatory biomarkers including IL-6, IL-8, MCP-1, VCAM-1, etc. (Fang et al., 2010). This provides a possible mechanism of how miR-10a regulates inflammatory cytokines in monocytes.

Based on our investigation, miR-10a was not the only microRNA with a significant change in the expression level. However, the level of miR-10a in both ECs and EVs was increased significantly, while miR-126 was increased significantly only in ECs on microgrooved PDMS membranes. A previous report (Asgeirsdottir et al., 2012) shows that miR-126 targets VCAM-1 in ECs to regulate the adhesion of ECs to monocytes. Therefore, miR-126 mainly functions in ECs but not in extracellular vesicles.

Another significant finding is that miR-10a made by ECs could be transferred to monocytes through EVs to modulate the expression of IL-6 in monocytes, which was demonstrated by the inhibition of EV secretion and miR-10a inhibition in ECs. This contact-independent EV transfer mechanism suggests a novel EC–monocyte signaling in the vascular microenvironment. These findings provide another explanation of how elongated ECs in the straight part of the arteries are anti-inflammatory and athero-protective.

This study not only unravels a new mechanism of EC–monocyte communication through EVs but also provides an insight into the biophysical regulation of cell–cell signaling in a vascular microenvironment. Our findings provide a rational basis for the design and the fabrication of micro/nano-materials that can be used to regulate EC and monocyte functions for vascular tissue engineering.

## Data Availability Statement

The datasets generated for this study are available on request to the corresponding author.

## Author Contributions

SL and YZ: conception or design of the work. JL, SG, XM, YT, and HW: acquisition of the data. JL, SG, YZ, and SL: manuscript writing. All authors have made significant contributions to the work, including analysis and interpretation of data, have approved the submitted version, and have agreed both to be personally accountable for the author’s own contributions and to ensure that questions related to the accuracy or integrity of any part of the work.

## Conflict of Interest

The authors declare that the research was conducted in the absence of any commercial or financial relationships that could be construed as a potential conflict of interest.
