# Supplementary material for: Endothelial Cell Morphology Regulates Inflammatory Cells Through MicroRNA Transferred by Extracellular Vesicles
Source: Front Bioeng Biotechnol. 2020 May 19;8:369. doi: 10.3389/fbioe.2020.00369 (PMC7248333; doi:10.3389/fbioe.2020.00369)
Supplement: Supplementary file 1 [file Data_Sheet_1.pdf]

Supplementary Figures

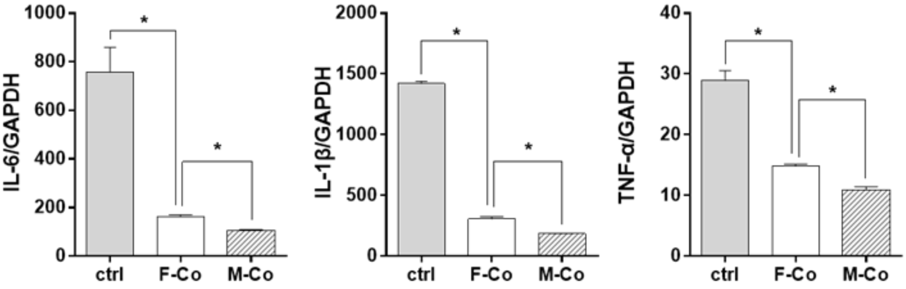

Figure S1. ECs on PDMS membranes can inhibit the inflammatory response of monocytes. The expression level of inflammatory cytokines (IL-6, IL-1β and TNF-α) was assessed by qRT-PCR. Data was normalized to GAPDH expression. The control group was LPS-stimulated monocytes in the absence of ECs. F-Co and M-Co are LPS-stimulated monocytes co-cultured with ECs on flat or microgrooved PDMS membranes, respectively. * $p<0.05$.

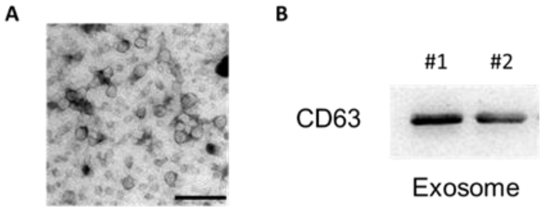

Figure S2. EV characterization. Exosomes were extracted from EC-conditioned medium. A, the TEM image of exosomes. Scale bar, 100 nm. B, the expression of the exosome marker CD63 was examined by Western-blotting analysis. Two parallel samples were examined, marked as #1 and #2.

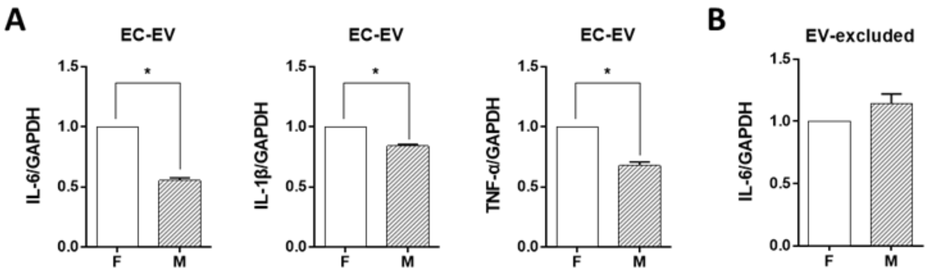

Figure S3 Morphology-modified ECs inhibited the inflammatory response of monocytes through EVs. EVs extracted from ECs on flat or microgrooved PDMS membranes and EV-excluded EC-conditioned medium was applied to culture monocytes. A and B, the expression level of inflammatory cytokines (IL-6, IL-1β and TNF-α) was assessed by qRT-PCR. Data was normalized to GAPDH expression. * $p<0.05$.
